# Computational Identification of Tissue-Specific Splicing Regulatory Elements in Human Genes from RNA-Seq Data

**DOI:** 10.1371/journal.pone.0166978

**Published:** 2016-11-18

**Authors:** Eman Badr, Mahmoud ElHefnawi, Lenwood S. Heath

**Affiliations:** 1 Department of Information Technology, Faculty of Computers and Information, Cairo University, Giza, Egypt; 2 Center of Excellence for Advanced Sciences, Informatics and Systems Department, National Research Center, Cairo, Egypt; 3 Center for Informatics Science, Nile University, Sheikh Zayed City, Egypt; 4 Department of Computer Science, Virginia Tech, Blacksburg, Virginia, United States of America; University of Miami, UNITED STATES

## Abstract

Alternative splicing is a vital process for regulating gene expression and promoting proteomic diversity. It plays a key role in tissue-specific expressed genes. This specificity is mainly regulated by splicing factors that bind to specific sequences called splicing regulatory elements (SREs). Here, we report a genome-wide analysis to study alternative splicing on multiple tissues, including brain, heart, liver, and muscle. We propose a pipeline to identify differential exons across tissues and hence tissue-specific SREs. In our pipeline, we utilize the DEXSeq package along with our previously reported algorithms. Utilizing the publicly available RNA-Seq data set from the Human BodyMap project, we identified 28,100 differentially used exons across the four tissues. We identified tissue-specific exonic splicing enhancers that overlap with various previously published experimental and computational databases. A complicated exonic enhancer regulatory network was revealed, where multiple exonic enhancers were found across multiple tissues while some were found only in specific tissues. Putative combinatorial exonic enhancers and silencers were discovered as well, which may be responsible for exon inclusion or exclusion across tissues. Some of the exonic enhancers are found to be co-occurring with multiple exonic silencers and vice versa, which demonstrates a complicated relationship between tissue-specific exonic enhancers and silencers.

## Introduction

Alternative splicing (AS) is an essential cellular process in eukaryotes that pre-mRNA usually undergoes to produce multiple mRNA isoforms of the same gene with likely different functions [1]. In a typical splicing process, introns within the pre-mRNA are removed, and the exons are joined together to form the mature mRNA [2, 3]. AS supports the joining of different combinations of exons; said another way, what sequence constitutes an exon is readily redefined. As a consequence, different proteins are produced from the same gene. Recent studies show that AS occurs in more than 95% of human genes [3, 4]. AS is regulated by specific proteins, called splicing factors. They bind to certain short sequences on the pre-mRNA, called splicing regulatory elements (SREs). Identifying these SREs and their combinatorial effects are crucial to understanding AS. SREs are classified as enhancers if they promote exon inclusion and silencers if they inhibit exon inclusion [3, 5, 6]. Accurate splicing is crucial, as it is believed that mutations either in the core splicing signals or in the SREs contribute to approximately 50% of human genetic diseases [4, 7, 8].

Alternative splicing plays a key role in generating tissue-specific proteins [3, 9]. Tissue-specific alternative splicing is regulated by a combination of tissue-specific and ubiquitously expressed RNA-binding factors [10]. They interact with the splicing regulatory elements to affect the spliceosome assembly (splicing machinery) and consequently the transcribed isoforms. There are several splicing factors that activate or repress splicing in different contexts [10]. Skipping exons is one of the notable alternative splicing events between different tissues that results in different proteins. Wang, et al. [10] suggested that having this switch-like regulation between tissues requires additional splicing regulatory elements to be present. Most work in mammalian systems revealed that AS decisions are often made by a combinatorial action of general and tissue-specific regulators [11]. Even simple tissue-specific decisions can involve additional layers of complexity, where regulatory elements cooperate or compete with each other [12].

Several studies have identified tissue-specific regulatory elements. Mouse RNA-Seq data for three tissues (brain, liver and skeletal muscle) were utilized to calculate the expression level of each isoform of genes for a set of predefined cassette exons [3]. The authors identified 456 putative enhancers and silencers. Among these, 45 were common to all tissues.

Kim et al. [13] utilized a distribution-based quantitative association rule mining to find exonic and intronic sequence motifs in 10 mouse tissues. Combinatorial *cis*-regulatory motifs were also discovered. The author identified statistically significant associations between sequence motifs and tissue-specific exon skipping rates. Ninety-seven interesting association rules were identified, of which three contain multiple 7-mers.

Focusing on human tissues, a varying effect regression model on splicing elements (VERSE) was developed to predict genome-wide intronic SREs [14], where RNA-Seq data for 16 human tissues was used. Approximately half of the SREs (55.68%) were found to be significant only in one tissue.

Castle, et al. [15] designed microarrays monitoring exons and exon-exon junctions in 17,939 human genes. For the regulatory elements, they extracted sequences in eight neighborhoods around regulated exons where 135 motifs were identified.

Brudno, et al. [16] identified intronic regulatory elements that are brain-specific, while, in [17], the identified regulatory elements were muscle-specific. In [8], a probabilistic approach was utilized and several intronic regulatory elements in different human tissues were identified.

Wang, et al. [18] developed a linear regression model to estimate the effect of various splicing factors on exon inclusion between two tissues, and, hence, the binding sites of these splicing factors are predicted. They applied their model on data from liver and heart tissues and predicted 15 motifs that contribute to exon skipping events. The work was extended to 11 human tissues in [9].

Wang, et al. [10], analyzed 10 human tissues. A high frequency of tissue-specific regulation was observed for each of various alternative splicing event types, including over 60% of the analyzed skipped exons.

Ke and Chasin [19] utilized a hypergeometric test to discover sequence pairs that are over-represented in intronic regions flanking human exons. They identified more than 60,000 5-mer sequence pairs with *p* ≤ 10^−4^. They showed that some pairs are associated with tissue-specific genes.

Taking into consideration the combinatorial effect of SREs, a biophysical principals based regression model for the regulation of AS was developed in [20]. It captures both the main effects of individual SREs and the combinatorial effects of SRE pairs. Overall, 619 different SREs and 196 SRE pairs were detected from different tissues. Their model was limited to the interaction of at most two SREs.

As discussed above, most of the human tissue-specific studies focus on identifying individual SREs in intronic regions [8, 14, 16, 17]. Wang, et al. [9, 18] identified SREs in both intronic and exonic regions. However, the focus was only on individual regulatory elements as well. There are other studies that identify individual SREs in both exonic and intronic regions [21–28], which we use for comparing our results. However, the focus in these studies was not on tissue-specific SREs. For more detailed review about these approaches, please refer to our previous paper [21].

The tissue-specific studies that focus on the combinatorial SRE effect were [19, 20]. In [20], out of 196 identified SRE pairs, only two pairs have both SREs in the exonic region. In [19], the identified pairs reside in the intronic regions.

In this work, we performed genome-wide analysis to study alternative splicing on multiple tissues (brain, heart, liver, and muscle). The RNA-Seq data set from the Human BodyMap project [29] was utilized. We used DEXSeq [30] to identify differential exons across tissues. Then, we applied our algorithms, GenSRE [21] and CoSREM [31], to identify both individual and combinatorial exonic regulatory elements responsible for exons that exist in one tissue but not in other tissues. Putative tissue-specific exonic enhancers were discovered and a complicated exonic enhancer regulatory network was revealed. Multiple exonic enhancers were found across multiple tissues, while some were found only in specific tissues. Putative combinatorial exonic enhancers and silencers were discovered as well that may be responsible for exon inclusion or exclusion across tissues. This is, to our knowledge, the first analysis to focus only on discovering exonic regulatory elements (individual and combinatorial) across tissues.

## Materials and Methods

### Data

RNA-Seq data from the Human BodyMap 2.0 project is utilized [29]. This data originates from 16 different human tissues. It contains 50 bp paired-end reads, 75 bp single-end reads, and 100 bp single-end reads. We focus on four tissues, namely, brain, heart, liver, and muscle. All the samples provided for tissues of interest have been utilized.

For comparing our results with previously published results, several databases are utilized. SpliceAid-F [22] is a recent comprehensive database that includes all the experimentally verified splicing factors and their binding sites. It contains 71 splicing factors and 655 binding sites for human. We also used AEdb [23], which is a database for alternative exons and their properties from various species; it is the manually curated component of the Alternative Splicing Database (ASD). The exon data in AEdb have been experimentally verified.

In addition, we compared our putative exonic splicing enhancers (ESEs) with five other computational data sets. The RESCUE-ESE [24] data set contains 238 6-mers for human exons. Another data set is PESE [25], where 2096 8-mers were identified. The third data set is from [26] and contains 4- and 5-mers as potential ESEs. In the data set from [27], the authors concentrated on 5-mer putative ESEs.

For exonic splicing silencers (ESSs), we compared our results with FAS-ESS [28], and PESS [25]. The FAS-ESS data set contains 130 10-mer sequences that were identified utilizing the mini-gene approach. PESS is another data set where the authors compared the frequencies of 8-mers (allowing one mismatch) in constitutively spliced non-coding exons with those in pseudo-exons and the 5′ untranslated regions (UTRs) of intronless genes.

### Overview of our proposed pipeline

**Fig 1:**
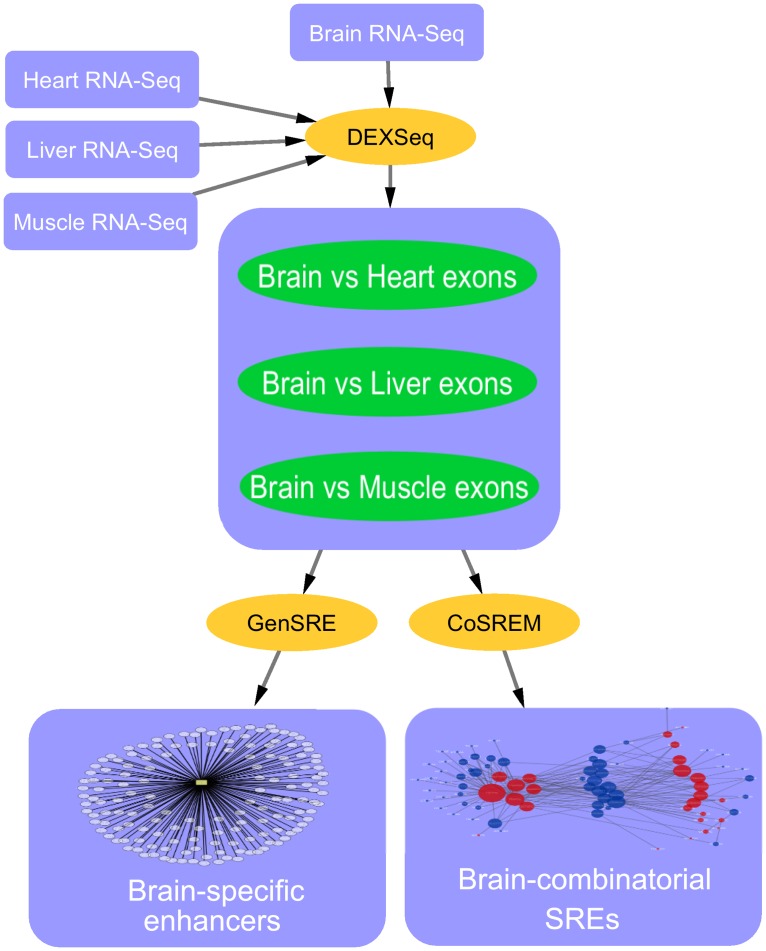
An example of the proposed pipeline applied on the brain tissue.

In our pipeline, different tools are utilized to identify tissue-specific exonic regulatory elements. The first stage (Fig 1) is to identify differential exons. To do that, we utilized DEXSeq [30]. DEXSeq identifies exons that are differentially used between two tissues. We compare each tissue of interest with the other three tissues. The output of this stage is three sets of differentially used exons in the tissue of interest but not in the remaining tissues. We then apply GenSRE [21] separately on the exon sets to identify exonic enhancers. We identify tissue-specific exonic enhancers by determining the common ESEs across the sets. We also apply CoSREM [31] to identify co-occuring exonic enhancers and silencers that may be responsible for exon inclusion or exclusion across tissues.

### Identifying differential exons across tissues

Our main goal is to identify splicing regulatory elements that are tissue-specific. To do that, we utilized DEXSeq [30] to identify exons that are differentially used between two tissues. Differential exon usage analysis aims at identifying the changes in relative usage of exons caused by a certain condition [32].

Let *R*_*g*_ = {*t*_1_, *t*_2_, …, *t*_*n*_} be the set of all the transcripts from a specific gene *g*. Let *X*_*g*_ = {*e*_1_, *e*_2_, …, *e*_*m*_} be the set of all exons that constitute the transcripts of gene *g*. Let *T*(*e*, *g*) be the set of all the transcripts from *R*_*g*_ that contain an exon *e*. Exon usage *U*(*e*, *g*) of exon *e* is defined to be

$$\begin{array}{ccc} U(e,g) & = & \frac{T(e,g)}{n} \end{array}$$

In DEXSeq, generalized linear models are utilized to model read counts, and the *χ*^2^ likelihood ratio test is then used to get an analysis of deviance *p*-value. The null hypothesis in this test is that none of the conditions influence exon usage. Rejecting the null hypothesis indicates that the count of sequencing reads that map to the exon under the test differs significantly between the different conditions. One of the advantages of this model is accounting for biological variability when the data has replicates for different conditions in contrast to other methods [33].

Before conducting differential exon usage analysis, flattening gene models and counting the reads were performed. Flattening gene models means aligning the sequencing reads to a reference genome and accumulating all the reads for each exon in each tissue from all the transcripts that contain this exon. As some of the transcripts may contain only a part of an exon, the *exon counting bins* term is used to refer to an exon or a part of an exon [32, 34]. It is notable that having exons with differential usage does not mean that their corresponding transcripts are differentially expressed. The output of DEXSeq is a table that contains the differential counting bins, their genes, their read counts in both tissues, and the *p*-values to determine significance.

For each pair of tissues, we applied DEXSeq. We identify differentially used exons by choosing the exons with *p*-value ≤ 0.05. We further filter these exons by choosing the exons with log_2_ fold change ≥ 2 or ≤ −2. To focus on exons that exist in one tissue but not in the other, we only used exons that have reads in one tissue and no reads at all in the other tissue. The output of this stage is a set of exons in each tissue that are differentially used and present in one tissue but not the other.

These exons were then retrieved from the ENCODE project [35]. The February, 2009, human genome assembly (GRCh37/hg19) was used. The complete exonic sequences were retrieved. The 200 intronic nucleotides upstream and the 200 intronic nucleotides downstream of each exon were also retrieved.

### Identifying tissue-specific enhancers

We utilized a formalism based on de Bruijn graphs that we previously reported [21]. It combines genomic structure, word count enrichment analysis, and experimental evidence to identify enhancers found in exons [21]. In this model, a six-dimensional de Bruijn graph is constructed *G* = (*V*,*E*) over the DNA alphabet *σ* = {*A*,*C*,*G*,*T*}, and each vertex is associated with its rank based on LEIsc (log of the enrichment index, scaled) scores from Ke et al. [36]. Then, a subset *U* ⊂ *V* is chosen to be the 400 vertices with the highest LEIsc values. An SRE graph *G*_*U*_ is then constructed and its weakly connected components are calculated. The algorithm GenSRE is then applied to each weakly connected component to determine a set of potential enhancers. Finally, these sequences are submitted to word count enrichment analysis.

### CoSREM

CoSREM (Combinatorial SRE Miner) is an algorithm for discovering combinatorial SREs that we reported in [31]. CoSREM is a two-level graph mining algorithm that is applied to the SRE graphs from [21] to identify co-occurring sets of SREs. The focus is on identifying sets of exonic splicing regulatory elements whether they are enhancers or silencers. Experimental evidence is incorporated through the SRE graphs to increase the accuracy of the results. The identified SREs do not have a predefined length, and the algorithm is not limited to identifying only SRE pairs.

### GO enrichment analysis

We utilized the command-line version of Ontologizer [37], with the goal of determining the enriched GO annotations of the genes that contain the identified enhancers that appears only in one tissue.

For each tissue, the genes, whose exons were identified as differential exons, are utilized as a background data set (population set). For each exonic splicing enhancer in a specific tissue, the exon data set is searched to allocate each splicing enhancer, and the corresponding gene set is identified to form the study set. GO annotation files gene_ontology_edit.obo and gene_association.goa_human were downloaded. GO enrichment analysis is performed using the Topology-Elim algorithm. The Westfall-Young Single Step multiple testing correction procedure is then applied.

We are interested in the biological process annotations. Therefore, we choose the biological process category with the minimum adjusted *p*-value, where we consider only terms with *p* ≤ 0.05 to be significant.

## Results

### Differentially used exons between tissue pairs

In this study, we analyze four tissues from the RNA-Seq data of the Human BodyMap project [29]. DEXSeq [30] is utilized to identify differentially used exons between pairs of tissues. Table 1 illustrates a part of the DEXSeq output for each pairwise comparison.

**Table 1:** An example of DEXSeq output for brain and heart tissues.

ID	stat	padj	brain	heart	log_2_ fold	count1	count2
chr8_ANK1-:E006	124.56	4.08E-26	4.39	20.56	2.23	5	357
chr10_ABLIM1-:E015	121.81	1.58E-25	3.62	18.91	2.39	1	507
chr10_ACBD7-:E003	7.75	0.04	8.47	0.36	-4.57	415	0
chr10_ADD3+:E015	68.33	3.04E-14	12.16	1.59	-2.94	144	0
chr10_CCSER2+:E006	19.46	0.00027	3.59	15.95	2.15	7	161
chr10_NEBL-:E017	394.064	5.60E-84	3.88	18.06	2.22	1	1601

The ID column lists the gene name and the exon number. The stat column includes the likelihood ratio test (LRT) statistic value. Brain and heart columns contain the exon usage coefficients for both tissues. The count columns include the actual counts of the mapped reads.

For each tissue pair comparison, DEXSeq produces a list of exons that are differentially used in one tissue but not in the other tissue. These exons may be whole exons or parts of exons. Being conservative, we define *tissue-unique exons* as differentially used exons with *p* ≤ 0.05, log_2_ fold change ≥ 2 or ≤ −2, and exons that have reads in one tissue and zero reads in the other tissue. We preferred to have a strict set of exons that exist in one tissue against the other. Table 2 illustrates the number of exons resulting from each pairwise comparison. The complete list is listed in S1 File.For example, applying DEXSeq on brain and heart tissues resulted in 1423 exons that are brain-specific. In the same manner, 857 exons were found to be heart-specific while having zero reads in brain tissue. It should be noticed that brain tissue has the largest number of tissue-unique exons, which is consistent with results reported in [13] that the brain has a large number of tissue-specific alternative spliced exons. We performed 6 pairwise tissue comparisons. Therefore, we have 3 different sets of exons for each tissue with a total of 12 tissue-unique exon sets for all 4 tissues.

**Table 2:** Number of tissue-unique exons that are present in one tissue and excluded in the other tissue.

	Brain	Heart	Liver	Muscle
Brain	-	1423	4592	7071
Heart	857	-	3952	6975
Liver	800	839	-	5002
Muscle	623	612	2495	-

The counts are tissue specific to the rows, while the columns show the second tissue in a comparison.

### Tissue-specific exonic enhancers

We used a de Bruijn graph based algorithm called GenSRE to identify ESEs in each tissue [21]. Having three exon sets for each tissue, we applied GenSRE on each set separately. Table 3 indicates the number of the identified ESEs in each set. The complete list is listed in S2 File. We focused on identifying putative exonic enhancers that may play a role in the inclusion of these exons within these specific tissues. In GenSRE, the exonic flank size is determined to be 50 nucleotides. Therefore, all exons that don’t meet this criterion are excluded. Table 4 contains the number of exons that were utilized. GenSRE identifies variable length SREs in the exonic flanking regions. The identified ESEs lengths range from 6 to 15 nucleotides.

**Table 3:** Number of identified ESEs in one tissue with respect to the other tissues using GenSRE algorithm.

	Brain	Heart	Liver	Muscle	Tissue-specific ESEs
Brain	-	449	793	923	205
Heart	250	-	695	877	85
Liver	277	398	-	752	50
Muscle	255	282	567	-	34

Tissue-specific ESEs are the common set between identified ESEs

**Table 4:** Number of utilized exons in GenSRE.

	Brain	Heart	Liver	Muscle
Brain	-	930	3132	4796
Heart	573	-	2638	4607
Liver	564	596	-	3404
Muscle	359	417	1634	-

To identify tissue-specific ESEs, we extracted the common set of exonic enhancers across the three exon sets for each tissue. This behavior suggests that these ESEs are tissue-specific as they repeatedly appeared in one tissue against all the other tissues. Table 3 illustrates the number of tissue-specific ESEs for brain, heart, liver, and muscle tissues. S1 Table includes the list of tissue-specific ESEs for all tissues.

**Fig 2:**
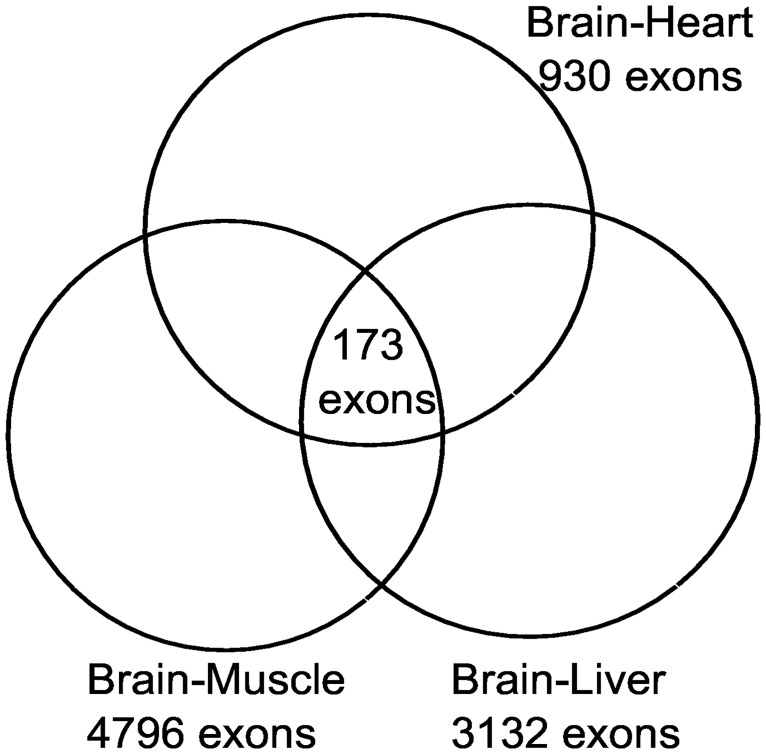
Brain-unique exons. Each circle represents the number of brain-specific exons that resulted from brain pairwise comparisons with other tissues (heart, liver, and muscle). The intersection represents brain-unique exons against all other tissues.

We also wanted to assess the accuracy of this pipeline in identifying tissue-specific ESEs. Therefore, for each tissue, we identified the set of exons that is present in only that tissue. This is done by finding the intersection between the different exon sets of each tissue. For example, in the case of the brain tissue, each exon set represents exons that are present in the brain and not in the heart, liver, and muscle tissues, respectively. The common set of exons between these three sets represents brain-unique exons (Fig 2).

Then, we searched for tissue-specific ESEs that we previously identified in these unique sets of exons, to see if they appear in the exonic flanking regions or not. Table 5 illustrates the number of ESEs that were found for each tissue. For example, out of 205 exonic enhancers that we identify as brain-specific ESEs, we found 173 ESEs in the brain-unique exons, which represents about 85% of all the identified ESEs. It is notable that, for instance, as indicated in Table 5 for the heart tissue, although there are 85 ESEs identified as heart-unique, the number of heart-specific exons is only 23 exons. In these 23 exons, we found 19 ESEs, which is about 83% coverage for the exons. This result suggests that our approach can be utilized to identify tissue-specific ESEs.

**Table 5:** A comparison table to identify the occurrence of tissue-specific ESEs in tissue-unique exon sets.

	Unique exons	Tissue-specific ESEs	ESEs found in unique exons
Brain	173	205	174
Heart	23	85	19
Liver	32	50	19
Muscle	21	34	13

Exons that are present in one tissue but not in all the other tissues.

### Comparison with SRE databases

We compared our results with databases from [22–27]. We extracted only exonic enhancers and silencers to compare our results with. We applied the same approach we used in [21].

We first compared our results with exonic binding sites from SpliceAid-F [22]. SpliceAid-F contains 330 different binding sited for human, 59 of them are exonic enhancers. Since our predicted ESEs are of variable length, as are SpliceAid-F binding sites, we calculated the overlap between the two sets by finding whether each sequence in the first list is entirely contained in at least one sequence in the second list or vice versa.

Another database is AEdb [23], where we considered only the 64 human enhancers.

In addition, we compared our ESE list with three other computational data sets, such as the RESCUE-ESE [24] data set, the PESE [25] data set, and the data set from [26]. The data set from [26] contains only 4- and 5-mers as potential enhancers. As a result, we could only test if sequences in our list include any of its sequences. This also applies to the data set from [27]. Table 6 summarizes the overlapping results. Overall, about 61.8% of the predicted ESEs can be matched to one of the previously published databases.

**Table 6:** Number of overlapped exonic enhancers with previously published data sets.

Data set	SpliceAid-F (69)	AEdb (64)	RESCUE-ESE (238)	PESE (2060)	Fedrove (42)	Zhang (42)	Total
Brain(205)	35	23	36	130	11	5	126 (61.5%)
Heart(85)	11	8	12	47	12	1	50 (59%)
Liver(50)	8	3	10	28	6	2	30 (60%)
Muscle(34)	9	6	6	22	3	3	25 (73.5%)

The numbers between brackets are the number of exonic enhancers in each database. The last column indicates the total number of identified ESEs from all previously published databases.

### Tissue-specific ESE regulatory network

**Fig 3:**
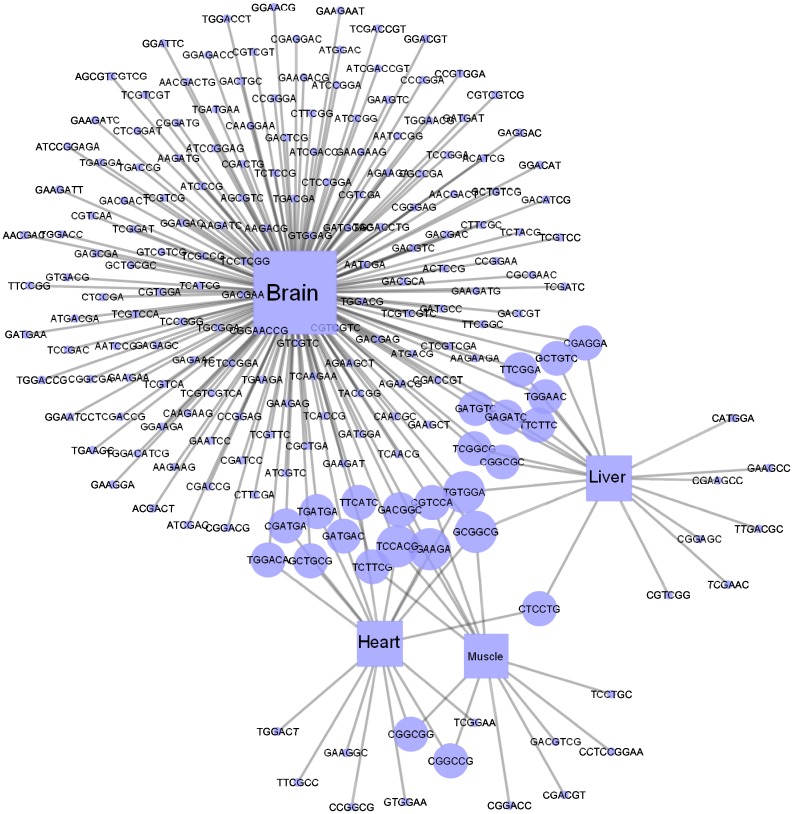
Tissue-specific ESE regulatory network. The circular nodes represent ESEs, and the rectangular ones represent tissues. An edge indicates an ESE contained in a tissue. The node size indicates the node degree.

To understand the complex relationship among the identified exonic enhancers found in multiple tissues, we constructed a regulatory network (Fig 3). It is a bipartite graph with two types of nodes: the circular nodes represent ESEs and the rectangular ones represent tissues. The size of any node type is proportional to its degree (number of incident edges). It is clear that there are ESEs that are specific to one tissue and others that may regulate more than one. That is in accordance with the suggestion in [11] that AS decisions are often made by a combinatorial action of general and tissue-specific regulators.

**Fig 4:**
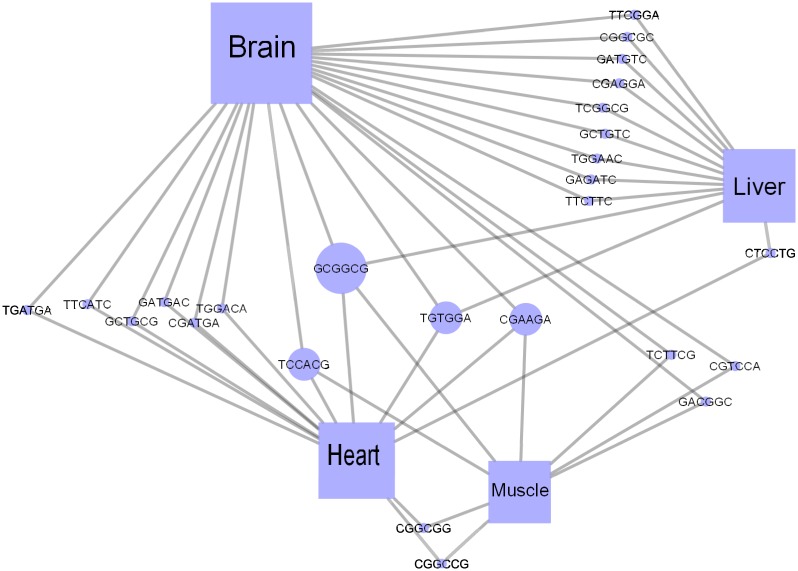
Enhancer regulatory network that focuses on enhancers that are involved in multiple tissues. The node size and color are proportional to its degree.

Focusing on the ESEs that are involved in multiple tissues (Fig 4), we noticed a hierarchical relationship, where some ESEs regulate two tissues (10% of all ESEs). A smaller number regulate 3 tissues (1%), and only one ESE is found in all four tissues. The other 89% of the SREs are tissue-specific where brain-specific exonic enhancers represent 64% of all the identified ESEs. These results are consistent with the conclusions from [13], that brain tissue exhibits a very large number of tissue-specific SREs and a limited number of general ones. However, in [13], the focus was on intronic SREs.

### Tissue-specific combinatorial SREs

Identifying individual *cis*-regulatory elements does not suffice to explain tissue-specific or condition-specific AS. For instance, in the case of exon skipping events, if an exon has both enhancer and silencer elements in proximity and in case of having an SR splicing factor with great affinity (SR factors are proteins that bind to enhancers and play various roles in spliceosome assembly [38]), the SR protein will bind to the enhancer and stimulate exon inclusion. This is through recruiting other spliceosome proteins, such as U1 and U2, to the core splicing signals. Consequently, the spliceosome machinery is assembled, and the exon is included.

On the other hand, if an inhibitory splicing factor such as hnRNP A1, which acts as a splicing repressor, is also present, it may inhibit the exon inclusion by binding to the silencer sequence and recruiting the binding of other inhibitory factors. These factors extend to the exon boundary and prohibit the binding of the SR protein. As a result, the exon will be skipped [11, 38].

We utilized our graph mining algorithm CoSREM [31] to identify co-occurring exonic enhancers (ESEs) and silencers (ESSs) that may cause exon inclusion in one tissue and its exclusion in another tissue. To do that, the identified exon set for each tissue was utilized. Table 7 illustrates the number of exons used for each tissue and the number of identified combinatorial exonic enhancers and silencers.

**Table 7:** Number of exons used in CoSREM and the resulted combinatorial SREs.

	No. of exons	Combinatorial SREs
Brain	8858	366
Heart	7818	283
Liver	4564	51
Muscle	2410	45

The actual combinatorial SRE sets are given in S3 File. We notice that these SRE sets appear in most of the specified exons. For example, in the brain tissue, the 366 combinatorial SRE sets appeared in 8753 out of 8858 exons, which may explain the inclusion of these exons in the brain tissue and their exclusion in the other tissues. Although the number of combinatorial enhancers and silencers in the brain tissue was quite large, the number of unique enhancers and silencers was surprisingly small. There are 63 unique ESSs and 30 unique ESEs, of which 26 are identified by GenSRE in the previous section as brain-specific ESEs. Table 8 illustrates the number of ESEs that were identified and verified previously.

**Table 8:** The number of overlapped ESEs with our previously identified sets by GenSRE algorithm.

	Brain(30)	Heart(14)	Liver(5)	Muscle(9)
No. of verified ESEs	26	9	4	1

The numbers in parentheses are the numbers of ESEs discovered as a part of a combinatorial set.

Table 9 illustrates the number of ESEs and ESSs identified as a part of combinatorial SREs for all the tissues.

**Table 9:** Number of ESEs and ESSs identified as a part of combinatorial SREs.

	No. of ESEs	No. of ESSs
Brain	30	63
Heart	55	55
Liver	15	24
Muscle	26	28

**Fig 5:**
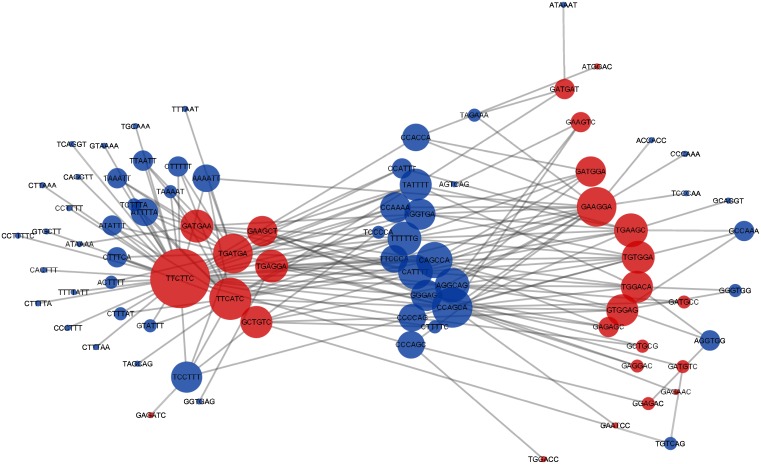
A regulatory network for combinatorial SREs identified in the brain tissue. The red nodes represent ESEs, and the blue ones represent ESSs. The node size is proportional to node degree.

To understand the relationship between these ESEs and ESSs, we constructed a regulatory network for exonic enhancers and silencers in the brain tissue (Fig 5). The red nodes represent ESEs, and the blue ones represent ESSs. The node size is proportional to node degree. The network illustrates the many-to-many relationship between the exonic enhancers and silencers. In other words, one ESE can co-occur with multiple ESSs and vice versa.

We wanted to assess the accuracy of the discovered ESSs. Our ESSs are compared with other data sets as illustrated in Table 10 such as SpliceAid-F [22], AEdb [23], FAS-ESS [28], and PESS [25],

**Table 10:** The number of overlapped ESSs with previously published data sets.

Data set	SpliceAid-F 59	AEdb 24	FAS 130	PESS 1091
Brain(63)	17	6	3	36
Heart(56)	16	5	3	37
Liver(24)	9	3	0	15
Muscle(28)	11	3	1	16

## Discussion

We propose a graph-based computational approach to identify exonic SREs that contribute to exon skipping events across tissues. We utilized DEXSeq [30] to identify differential exons across tissues, and then we applied GenSRE [21] and CoSREM [31] algorithms to identify both individual and combinatorial SREs that are tissue-specific. We utilized Ontologizer [37] to assess the significance of our predicted tissue-specific ESEs and whether they are involved in tissue-specific biological processes. Therefore, for each tissue, we determined the enriched GO annotations of the genes that contain the identified ESEs. We focused on identifying significant biological processes with adjusted *p*-value ≤ 0.05. We identified several brain-related processes, as illustrated in Table 11. We also identified some heart-related processes such as “regulation of cardiac muscle contraction by regulation of the release of sequestered calcium ion” with *p*-value 0.00295. Other biological processes were identified as liver and muscle-related such as “digestive tract morphogenesis”, and “regulation of muscle system process” with *p*-values 0.00202, and 0.00253, respectively. S4 File includes the complete list of the enriched GO annotations for the four tissues of interest. We applied the same analysis to identify the enriched GO annotations of the genes that contain combinatorial SREs. Various functionally related biological processes to the tissue of interest are recognized. The complete list of the enriched GO annotations of the four tissues is included in S5 File.

**Table 11:** Examples of biological processes that are brain and nervous system-related.

Enhancer element	ID	Annotation	*p*-value	Pop. count	Study total	Study count
CGGAAGA	GO:0042428	serotonin metabolic process	0.00027	3	49	2
TCGGAT	GO:0021553	olfactory nerve development	0.00028	2	86	2
AATCGA	GO:0048708	astrocyte differentiation	0.0003	13	54	3
ATGACGA	GO:0060291	long-term synaptic potentiation	0.00031	5	29	2
CGTCGT	GO:0001505	regulation of neurotransmitter levels	0.00036	30	59	4
GGAGAC	GO:0046928	regulation of neurotransmitter secretion	0.00049	4	257	3
GAGAGC	GO:0021983	pituitary gland development	0.0007	9	262	4
CGTCGAC	GO:0090210	regulation of establishment of blood-brain barrier	0.00216	1	11	1
ATGACG	GO:0007212	dopamine receptor signaling pathway	0.00248	5	82	2
TTCGGAT	GO:0007269	neurotransmitter secretion	0.00384	20	24	2
ACCGGGA	GO:0007269	neurotransmitter secretion	0.00718	20	33	2

They resulted of GO enrichment analysis of gene sets that contain putative brain-specific ESEs identified by our approach. Population total is the total number of genes in the population set. In this case, we have 5096 genes in the population set. Pop. count is The number of genes in the population set that are annotated to the GO term in question. Study total is the number of genes in the study set. Study count is the number of genes in the study set that are annotated to the GO term in question.

We also identified tissue-specific combinatorial SREs. These are sets of co-occuring exonic enhancer and silencer elements in each tissue. As our focus here is exons that are differentially used in one tissue with respect to other tissues, identified combinatorial SREs may play a role in exon inclusion in one tissue and its exclusion in another tissue. Mayeda et al. [39] showed *in vitro* that having different ratios of SF2/ASF to hnRNP A1 splicing factors promotes exon skipping or inclusion by binding to different enhancers or silencers. In other words, it was shown that the ratio of SF2/ASF to hnRNP Al can affect whether the internal exon is included or excluded. An excess of SF2/ASF promotes exon inclusion, while hnRNP Al excess promotes exon exclusion.

**Fig 6:**
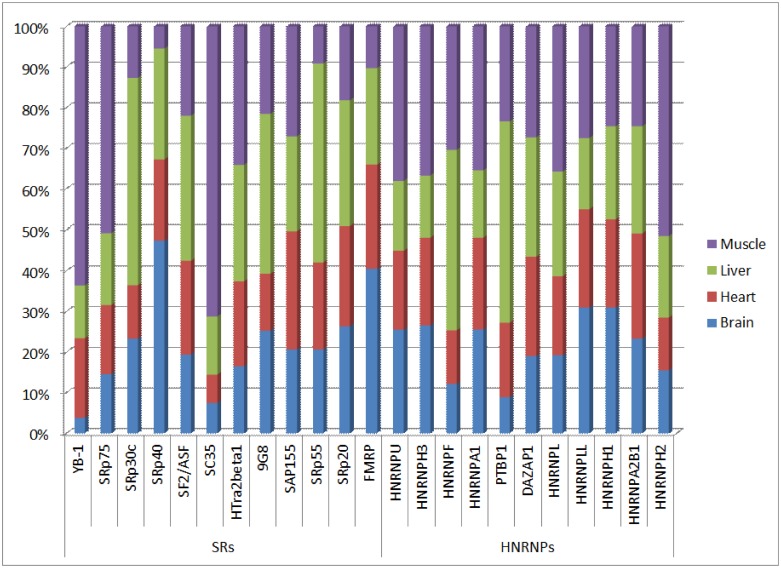
Relative expression levels for several splicing factors across the tissues from the RNA-Seq data. Relative expression level for a splicing factor in a specific tissue is calculated by dividing its expression level value over the sum of all expression level values for this splicing factor across the tissues. This shows the different abundances of splicing factors across tissues.

Therefore, we investigated this hypothesis by incorporating the splicing factor information. We identified splicing factor proteins (enhancer factors and inhibitor factors) from SpliceAid-F [22] that bind to SREs in our combinatorial SRE sets. We then calculated the expression levels for these splicing factors from the RNA-Seq data (Fig 6). The actual expression levels in FPKM is in S2 Table. Then, for each combinatorial SRE set, we calculated the ratio between the expression levels of its splicing factors, if any. Table 12 provides an example from the combinatorial SREs identified in the brain tissue. We focused on results where the ratio ≥ 1 in one tissue and ≤ 1 in at least another tissue, which suggests that these splicing factors and their binding sites may play a role in regulating exon inclusion or exclusion between the tissues.

**Table 12:** Identifying splicing factors that binds to combinatorial SREs in the brain tissue.

Combinatorial SRE set	Enhancer factor	Inhibitor factor	Brain ratio	Heart ratio	Liver ratio	Muscle ratio
CAAGGA, CAGCCA	FMRP	hnRNPLL	1.831381681	1.484036726	1.899282779	0.521982767
TGTGGA, CCAGCA	SRp55	hnRNPLL	1.706009449	2.279634755	7.114715878	0.846707243
TGTGGA, CAGCCA	SRp55	hnRNPLL	1.706009449	2.279634755	7.114715878	0.846707243
TGTGGA, TCCTTT	SRp55	DAZAP1	1.162119086	0.935388956	1.786036684	0.356244555
GAAGGC, AGGCAG	9G8	non	-	-	-	-
AGAAGAT, TTAGAA	9G8	non	-	-	-	-
GAAGGA, GGGAGG	HTra2beta1	hnRNP F	0.113064576	0.131103394	0.053528796	0.09326604
TTCTTC, TCCTTT	non	hnRNPA2B1	-	-	-	-
GAGGAT, GGGAGG	SF2/ASF	hnRNPA1	0.064157386	0.086308919	0.183388318	0.052519222
GAGGAT, GGGAGG	SF2/ASF	hnRNPF	0.697677691	0.761178655	0.350434389	0.316325285

The ratio columns contain the ration between the expression level of the enhancer factor and the inhibitor factor in the specified tissue. The cells with ‘non’ indicates that we could not identify an associated splicing factor and hence no ratios are provided.

An interesting result involved the splicing factors FMRP and hnRnpLL where the FMRP to hnRNPLL expression level ratio was (≈ 1.83) in brain tissue while it was (≈ 0.52) in muscle tissue as illustrated in Table 12. More interestingly, These splicing factors were associated with multiple SRE sets. We identified multiple co-occuring exonic enhancer and silencer elements that all are potential binding sites to these splicing factors in the brain tissue. For example, one ESS identified as a potential binding site to hnRNPLL protein is *CAGCCA*. It co-occurs with different exonic enhancer elements (*AAGAGA*,*AAGGAA*,*CAAGGA*,*GAGAGC*,*GTGGAG*, *AGAGGA*). All ESEs were identified as binding sites to FMRP splicing factor. Other ESSs elements were identified as well as binding sites to hnRNPLL such as *CCACCA*, *CCAGCA*. All the identified binding sites include *CA* dinucleotide repeats, which is known to be preferentially recognized by hnRNPLL [40]. This suggests the hypothesis that these two splicing factors may have an antagonistic behavior that results in some exons being included in the brain tissue and excluded in the muscle tissue. Although this hypothesis needs further experimental validation, our approach can highlight interesting results for more experimental testing.

## Conclusion

In summery, we report a genome-wide analysis to study alternative splicing on multiple tissues. That includes brain, heart, liver, and muscle tissues. Our proposed pipeline identifies differentially used exons across tissues and hence tissue-specific exonic SREs. Both individual and combinatorial SREs are identified which may be the reason for exon inclusion or exclusion across different tissues. Our approach helps understand the relationship between different types of exonic SREs. It yields interesting results that can open new directions to study alternative splicing and how it may influence tissue specificity.

## Supporting Information

S1 File It contains 6 tables. Each table contains a list of genomic coordinates of significant differential exons for each pairwise comparison between tissue pairs.
(XLSX)

S2 File It contains putative enhancers and its associated information for each pairwise comparison between tissue pairs. (XLSX)

S1 Table It includes the complete list of tissue-specific enhancers for all four tissues. (XLSX)

S3 File The file includes the complete list of combinatorial enhancers and silencers for the four tissues. (XLSX)

S4 File It includes the complete list of enriched GO annotations of genes with putative tissue-specific enhancers for all tissues. (XLSX)

S5 File It includes the complete list of enriched GO annotations of genes with putative tissue-specific combinatorial SREs for all tissues. (XLSX)

S2 Table It includes the expression levels for all splicing factors of interest. (XLSX)
